# Cyanidin-3-*O*-glucoside Regulates the Expression of *Ucp1* in Brown Adipose Tissue by Activating Prdm16 Gene

**DOI:** 10.3390/antiox10121986

**Published:** 2021-12-14

**Authors:** Suping Han, Yafan Yang, Yanan Lu, Jielong Guo, Xue Han, Yunxiao Gao, Weidong Huang, Yilin You, Jicheng Zhan

**Affiliations:** 1 Beijing Key Laboratory of Viticulture and Enology, College of Food Science and Nutritional Engineering, China Agricultural University, Tsinghua East Road 17, Beijing 100083, China; hansuping@cau.edu.cn (S.H.); S20213061075@cau.edu.cn (Y.Y.); luyanan@bcu.edu.cn (Y.L.); b20183060515@cau.edu.cn (J.G.); hanxuehx313@bjmu.edu.cn (X.H.); s20193060991@cau.edu.cn (Y.G.); weidonghuang@cau.edu.cn (W.H.); 2 School of Biomedicine, Beijing City University, Beijing 100094, China; 3 Department of Physiology and Pathophysiology, School of Basic Medical Sciences, Peking University Health Science Center, Beijing 100191, China

**Keywords:** brown adipose tissue, Cyanidin-3-*O*-glucoside, PR domain containing 16, *Ucp1* promoter, peroxisomes proliferator-activated receptors

## Abstract

(1) Background: Brown adipose tissue (BAT) burns energy to produce heat. Cyanidin-3-*O*-glucoside (C3G) can then enhance the thermogenic ability of BAT in vivo. However, the mechanism by which C3G regulates Ucp1 protein expression remains unclear. (2) Methods: In this study, C3H10T12 brown adipose cells and db/db mice and mice with high-fat, high-fructose, diet-induced obesity were used as the model to explore the effect of C3G on the expression of the *Ucp1* gene. Furthermore, the 293T cell line was used for an in vitro cell transgene, a double luciferase reporting system, and yeast single hybridization to explore the mechanism of C3G in regulating Ucp1 protein. (3) Results: we identified that, under the influence of C3G, Prdm16 directly binds to the −500 to −150 bp promoter region of *Ucp1* to activate its transcription and, thus, facilitate BAT programming. (4) Conclusions: This study clarified the mechanism by which C3G regulates the expression of the *Ucp1* gene of brown fat to a certain extent.

## 1. Introduction

Anthocyanins are polyphenolic compounds that are abundant in dark-colored fruits, vegetables, and pigmented cereals (such as berries, cherries, grapes, purple onions, black beans, purple cabbage, black rice, red sorghum, and purple maize (In addition, anthocyanins are commonly processed as colorants in beverages, fruit fillings, snacks, and dairy products, which account for a considerable amount of the anthocyanins consumed in the average diet [1]. Anthocyanin is a common component in functional foods for preventing cardiovascular diseases and inflammatory diseases (including diabetes and metabolic syndrome), mainly due to its excellent antioxidant activity [2]. In anthocyanin-related structures, the carboxypyranoside -3-*O*-glucoside, in particular, exhibits a good inhibition effect on active oxygen generation and high structural stability [3]. A large number of studies have proven that anthocyanins offer a variety of therapeutic effects, including that of increased energy expenditure and of limiting weight gain provided by Cyanidin-3-*O*-glucoside (C3G) [4].

The rising prevalence of excess body mass and obesity in nearly all countries around the world has been described as a global pandemic [5]. Currently available anti-obesity strategies are largely dependent on limiting energy uptake and/or absorption, but these tend to have little effect and often cause unwanted side effects [6,7]. An alternative strategy is urgently required to increase the energy expenditure in key metabolic organs, such as brown adipose tissue (BAT) [8]. In rodents, increasing BAT activity can effectively enhance metabolic rate and protect the animal from obesity [9].

BAT plays an essential role in non-shivering thermogenesis in mammals. Its cells are rich in mitochondria, the inner membranes of which are, in turn, rich in uncoupling protein 1 (Ucp1). BAT can specifically overexpress Ucp1, thus uncoupling transmembrane electron transfer and oxidative phosphorylation in the mitochondrial respiratory chain, thereby releasing a large amount of energy in the form of heat [10]. The known anti-obesity effect of functional BAT has drawn research and attention to the activation and regulation mechanism of brown fat functioning, and the specific expression of Ucp1 by BAT has become a hot topic for scientists.

The expression of the *Ucp1* gene is regulated by a variety of transcription factors, such as peroxisome proliferator-activated receptors (PPARs), PR domain containing 16 (Prdm16), peroxisome proliferator-activated receptor γ coactivator-1(PGC-1), and mitochondrial transcription factor A(TFAM). PPARα participates in the regulation of fatty acid oxidation mainly by inducing the expression of genes related to fatty acid oxidation [11]. Prdm16 is a zinc finger structure transcription factor containing PR domain, which is highly expressed in mouse BAT. It is also a key factor in determining whether adipose precursor cells develop into skeletal muscle cells or brown adipose cells. The Prdm16 protein is greatly enriched in BAT and causes increased expression of mitochondrial genes and greater density of mitochondria [12]. The loss of Prdm16 can hinder the differentiation of brown adipocytes, while overexpression can significantly increase their numbers. Animals lacking Prdm16 in BAT have a dramatically reduced capacity to produce heat [13]. Prdm16 regulates the thermogenic function of brown fat, mainly by binding to PPARγ and coactivating its transcriptional function [14].

The anti-obesity and anti-diabetic effects of anthocyanins are well-accepted experimentally [15,16]. Anthocyanins C3G and C3R have been reported to increase the mitochondrial copy number in vitro and to promote the metabolism of carbohydrates and fat [17]. C3G can enhance whole-body metabolism by upregulating the mitochondrial function of BATT and beige formation in subcutaneous WAT [4]. However, its molecular targets have yet to be elucidated.

Here, via quantitative real-time PCR, to quantify the expression of BAT-related genes *Ucp1* and *Prdm16* following the treatment of C_3_H_10_T_1/2_ cells with C3G during brown adipogenesis, we speculate that *Prdm16* may be a novel transcriptional activator of *Ucp1*. The results of a luciferase assay and yeast one-hybrid screening ascertained that C3G activates the thermogenic gene *Ucp1* by regulating the expressions of *Prdm16*. Thus, Prdm16 can bind directly to the *Ucp1* promoter region, from −500 to −150 bp to active transcription. Our results, thus, suggest that Prdm16 is indeed a novel transcriptional activator of Ucp1 and that C3G can increase energy metabolism through a previously unrecognized molecular mechanism. Consequently, these findings may support an innovative approach to the treatment of obesity and its related diseases.

## 2. Materials and Methods

### 2.1. Materials and Reagents

C3G (purity 95.7%) was purchased from Chengdu Manchester Co., Ltd. (Chengdu, China), A ready-to-use PCR kit 3.0 was purchased from Beijing Tianenze Biotechnology Co., Ltd. (Beijing, China), which is used for PCR identification of bacterial liquid, and the method is carried out according to the instruction manual. Dimethyl sulfoxide (DMSO), a double luciferase reporter gene detection kit, and Lipo8000TM transfection reagent were purchased from Beyotime Biotechnology Co., Ltd. (Shanghai, China), while a HiFi Script cDNA Synthesis Kit, Ultra SYBR Mixture, and 200 bp Ladder DNA Marker were purchased from Kangwei Century Biology Science and Technology Co., Ltd. (Beijing, China), and a mass extraction and purification kit for plasmid was purchased from Weigela Biology Technology Co., Ltd. (Chengdu, China). Furthermore, recombinant human PPARA (P0645) was purchased from Wuhan Fine Biotechnology Co., Ltd. (Wuhan, China).

### 2.2. Cell Culture

The mouse mesenchymal stem cell line, known as C_3_H_10_T_1/2_ mouse embryonic fibroblasts, was purchased from the cell bank of the Chinese Academy of Sciences (Shanghai, China), and was induced to differentiate into brown adipocytes. The C_3_H_10_T_1/2_ cells used in this experiment were cell samples frozen in liquid nitrogen in the laboratory.

The cryovials were taken out of the liquid nitrogen and immediately melted in a 37 °C water bath, centrifuged at 500× *g* for 3 min, and cultured in a 37 °C, 5% CO_2_ incubator for passage. When the cells grew to a density of 80–90%, the medium was changed to brown adipose differentiation medium (DMEM HIGH + 10% FBS + 0.02 μM INSULIN + 1 × 10^−3^ μM T3) and an appropriate concentration of C3G was added. Two days later (recorded as day 0), the medium was changed to brown fat induction medium (DMEM HIGH + 10% FBS + 0.02 μM INSULIN + 1 × 10^−3^ μM T3 + 0.125 mM INDOMETHACIN + 0.5 mM IBMX + 2 μg/mL DEX) and an appropriate concentration of C3G was added. Two days later (recorded as day 2), the medium was changed to the brown adipose differentiation medium described above, and an appropriate concentration of C3G was added. The liquid was changed again on the 4th day. On the sixth day, the cells were observed under an inverted microscope. There were obvious fat droplets in the cytoplasm, i.e., the differentiation was completed.

### 2.3. Animals Studies

Animal experiments were conducted according to the methods reported by You et al. [4,18]. Animal experiments were conducted in two stages. Firstly, 20 three-week-old obese C57BLKS/J-Leprdb/Leprdb (db/db) male mice (mouse model of hereditary obesity) were purchased from the Model Animal Research Center of Nanjing University, China. After an acclimation of one week, the mice were randomly divided into two groups (*n* = 10 per group) with overall equal body weights. The mice received C3G (1 mg/mL) dissolved in drinking water for 12 weeks. DMSO was used as the vehicle for different treatments. In addition, a further 36 three-week-old male C57BL/6J mice (obesity model mice induced by high fat and high glucose diet) were purchased from Vital River Laboratory Animal Technology Co., Ltd., China. After one week of acclimation, these mice were randomly divided into three groups (*n* = 12/group) with overall equal body weights. They were assigned to one of three dietary treatments for 12 weeks: (1) normal chow diet group (CHOW, 3.2 kcal/g, 4.5% fat, *w*/*w*); (2) diet-induced obesity group (DIO, 4.7 kcal/g, 25% fructose and 25% lard); and (3) C3G group (DIO + C3G, 4.7 kcal/g, 25% fructose and 25% lard). C3G-group mice received C3G dissolved in drinking water (1 mg/mL), while drinking water also served as the vehicle for the CHOW and DIO treatments. Three mice were housed per cage in an Office of Laboratory Animal Welfare-certified animal facility, with a 12-h light/12-h dark cycle. Principles of laboratory animal care were followed, with all procedures conducted according to the guidelines established by the National Institute of Health, and every effort was made to minimize suffering.

### 2.4. Isolation of Total RNA Extraction and Analysis by qPCR

The total RNA of cells and BAT were extracted using RNAiso Plus reagent. Isolated RNA was quantified by measuring optical density (OD) at 260 and 280 nm with a NanoDrop 2000 spectrophotometer (Thermo Scientific, Waltham, MA, USA). The cDNA was synthesized by means of a PrimeScript RT reagent kit (Takara Biotechnology Co., Ltd., Dalian, China) and the assay was performed using real-time PCR with a SYBR Premix Ex Taq^TM^ I and 7500 Real-Time PCR System (Applied Biosystems, Foster City, CA, USA). Data were normalized to the internal control actin and analyzed using the ΔΔCt method. Primer sequences for the *Ucp1* promoter and *Prdm16* are listed in Table 1.

### 2.5. Construction of Plasmid Vector

The primers were designed and cloned according to the selected gene fragments, and the target gene was recombined with the vector and transformed into *E. coli*. Thereafter, PCR and double enzyme digestion were performed, followed by sequencing for identification, and the detected correct plasmid vector was used for subsequent experiments. The cleavage sites and vector plasmids of the different gene fragments involved in this experiment are shown in Table 2.

The primer sequence was designed by CE Design, via the official website of Nanjing Vazyme Biotechnology Co., Ltd. (Nanjing, China), (www.vazyme.com (accessed on 31 May 2020)), according to the gene sequence provided by NCBI, and the primer was synthesized by Shanghai Sangon Biological Engineering Technology Co., Ltd. (Shanghai, China).

### 2.6. Cell Transfection and Luciferase Activity Detection

Cell transfection was performed using the method described in the Lipo8000 Transfection Reagent, Beyotime Biotechnology, Inc., with some modification. The transfection reagent was configured to 50 µL of Opti-MEM^®^ (Thermo Scientific, Waltham, MA, USA) Medium, 1 µg of plasmid DNA, and 1.6 µL of Lipo8000™ (Beyotime Biotechnology, Shanghai, China) transfection reagent. After 24 h transfection, the cells were lysed, and firefly luciferase detection reagent and renal luciferase detection working solution were added, respectively, to measure RLU on a multifunctional microplate reader.

### 2.7. Yeast One-Hybrid Assay

In order to verify the interaction between *Prdm16* and the *Ucp1* promoter, in this experiment, the *p*LacZi-Ucp1 reporter vector and *p*JG-Prdm16 fusion expression vector were constructed and transfected into yeast to screen for blue leukoplakia.

Respectively, the *Prdm16* was connected with the PJG plasmid after enzyme digestion, the *Ucp1* promoter was connected with the pLacZi plasmid after enzyme digestion, and co-transfection was performed into EGY48 competent cells. The cells transfected with Placezi+ and PJG+ were used as the positive control, *p*LacZi-Ucp1 and PJG empty vectors were used as the self-activated control, and the Placezi empty vector and *p*JG-Prdm16 were used as the negative control. The cells were cultured in a SD-trp/ura-deficient medium for 48 h, after which single colonies were picked and cultured in X-α-gal medium for 48 h, and the results were observed.

### 2.8. shRNA-Mediated Gene Silencing of Prdm16

According to the gene sequence number information provided by NCBI, a primer sequence was designed through the GPP Web Portal website: 5′-CCGGGACGGTGACGTTGTAAATAATCTCGAGATTATTTACAACGTCACCGTCTTTTTG-3′. The pLKO.1 vector was enzymatically cleaved with AgeI (NEB, #R0552S) and EcoRI (NEB, #R0101S) for ligation and transformation. The collected plasmids were transferred into C_3_H_10_T_1/2_ cells and inoculated onto a 6-well cell culture plate. After further differentiation for 6 days, according to the brown adipose differentiation method, the mRNA expressions of prdm16 and Ucp1 mRNA the cells were detected by RT-PCR.

### 2.9. Microscale Thermophoresis (MST) Study

The MST micro-thermophoroscope (Monolith NT.115) was derived from Northrop. First, the PPARα protein was fluorescently labeled to make it become a reporter molecule, and the gradient-diluted C3G molecule was mixed with the PPARα protein of the same concentration in an equal amount and loaded into the capillary. The affinity of molecular interactions was quantitatively analyzed by detecting the fluorescence changes caused by thermophoresis in capillaries with different concentrations under the temperature gradient field. The experimental results were automatically analyzed by MO Affinity Analysis software, and the Kd value was calculated accurately.

### 2.10. Analysis of Experimental Data

All data were expressed as means ± SD. Statistical analysis was conducted using GraphPad Prism (version 7.0). The statistical differences between the groups were evaluated using one-way ANOVA with a least significant difference (LSD) test, and a *p*-value less than 0.05 was considered statistically significant.

## 3. Results

### 3.1. C3G Upregulates the Expression of Ucp1 Gene Both In Vitro and In Vivo

**Figure 1:**
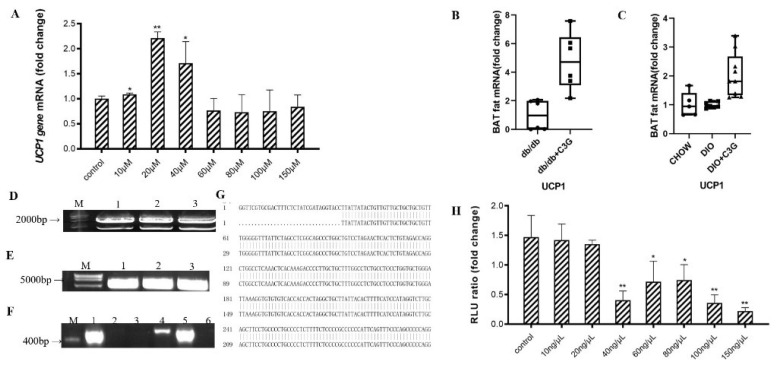
C3G was able to up-regulate the expression of *Ucp1* gene both in vitro and in vivo. (**A**) Effects of C3G at different concentrations on *Ucp1* gene expression in C_3_H_10_T_1/2_ cells. (**B**,**C**) C3G up-regulated the expression of *Ucp1* gene both in db/db mice model (**B**) and mice with DIO model (**C**) (*n* = 6–8). (**D**) Electrophoretic map of cloned Ucp1, M: Kang was 200Bpladder; Lane 1–3: Ucp1 amplification products (**E**) Electrophoresis diagram of pGL3-Basic enzyme digestion product, M: DL15000 marker; Lane 1–3: pGL3-Basic double enzyme digestion product.(**F**) PCR electrophoresis diagram of pGL3-Ucp1 bacterial solution; M: Kang Wei 200Bpladder; Lane 1–6: PCR products from the picked six single-colony bacterial solutions.(**G**) Comparative diagram of sequencing results of PGL3-Ucp1 (**H**) Activation of *Ucp1* promoter by C3G with different concentrations. Values represent means ± SD. Error bars represent SD; mean values with asterisk or different letters are significantly different (* means *p* < 0.05; ** means *p* < 0.01).

As mentioned above, it has previously been reported that C3G treatment specifically upregulates the thermogenic gene expressions in BAT-cMyc cells [17]. In this study, we treated C_3_H_10_T_1/2_ cells with various concentrations of C3G to explore the effect of C3G on the expression of the *Ucp1* gene. However, this proved to have no effect on BAT-cMyc cell viability or proliferation during brown adipogenesis. In comparison with the PBS medium, C3G at concentrations of 10 μM, 20 μM, and 40 μM was found to significantly upregulate the relative expression level of *Ucp1* mRNA in the C_3_H_10_T_1/2_ cells (*p* < 0.05) and, at 20 μM, the C3G could significantly upregulate the expression of Ucp1 (Figure 1A) (*p* < 0.01). Similarly, through the animal experiments, it was found that C3G treatment could significantly increase the expression of the *Ucp1* gene of BAT in db/db mice and mice with DIO. The expression of *Ucp1* in the C3G-treated mice was 3.82 times and 3.06 times higher than those in the two control groups (Figure 1B,C). These results are consistent with our previous research results [4,17], further demonstrating C3G-induced upregulation of *Ucp1* mRNA expression, both in vitro and in vivo.

All figures and tables should be cited in the main text as Figure 1, Table 1, etc.

Next, we successfully constructed the luciferase reporter gene vector (Figure 1D–G), to examine the role of C3G in the activation of *Ucp1*. The luciferase assay showed that, compared with the PBS-supplemented medium, treatments with various concentrations of C3G and transfection with the −2.0 kb *Ucp1* promoter–luciferase did not activate the *Ucp1* promoter (Figure 1H) (*p* < 0.01). Thus, these results indicate that, while C3G significantly accelerates *Ucp1* expression, it does not directly activate its promoter.

### 3.2. C3G Upregulates the Expression of Prdm16 Gene Both In Vitro and In Vivo

**Figure 2:**
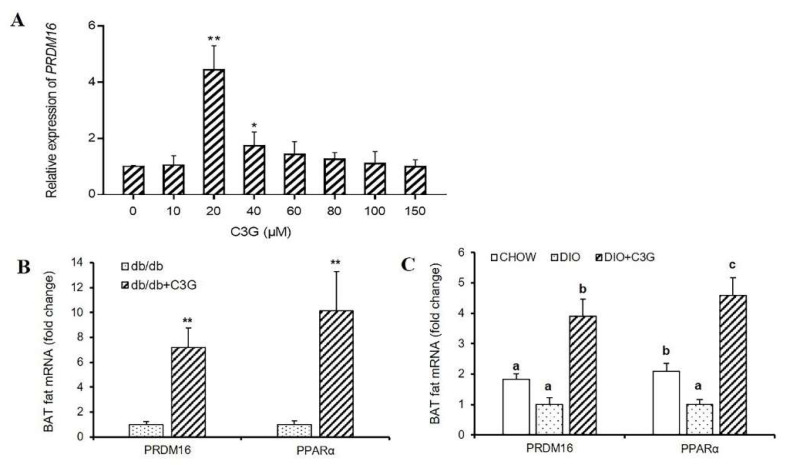
C3G was able to up-regulate the expression of *Prdm16* gene both in vitro and vivo. (**A**) Effect of C3G at different concentrations on *Prdm16* gene expression in C3H10T12 cells. (**B**,**C**) Gene expression profile in BAT, real-time PCR analysis of thermogenic-related genes, including *Prdm16*, PPARα in db/db mice model (**B**) and mice with DIO model (**C**) (*n* = 6–8). Values represent means SD. Error bars represent SD; mean values with asterisk or different letters are significantly different (* means *p* < 0.05; ** means *p* < 0.01).

As the expression level of the BAT-specific gene *Ucp1* was increased by C3G treatment and this process was not dependent on the activation of the *Ucp1* promoter, we speculated the existence of other regulators as target genes of C3G, such as transcription factors. *Prdm16* determines the brown fat-like program and thermogenesis in both brown and white adipose tissues [19,20,21,22], so we consequently sought to determine whether this thermogenesis-related gene participates in this process, and found that treatment with C3G (20 μM) resulted in a 4.42-fold increase in *Prdm16* mRNA expression in C_3_H_10_T_1/2_ cells, compared to the control group. Interestingly, the expression levels of *Prdm16* followed the same trend as that of *Ucp1* after treatment with various concentrations of C3G (Figure 2A) (*p* < 0.01). Moreover, the promotional effect of C3G on *Prdm16* was consistent with that in previous reports [4,17]. Similarly, in light of the animal experiments in this study, it was ascertained that C3G treatment can significantly increase the expression of *Prdm16* and *PPARα* genes in the BAT of mice. In the db/db mouse model and the DIO model mice, the expressions of *Prdm16* and *PPARα* genes in C3G-treated mice increased by 7.21 times, 10.16 times, 3.91 times, and 4.58 times (*p* < 0.05), respectively, compared with the control group (Figure 2B,C). These results are consistent with our previous research results [4,17], indicating clearly that C3G treatment increased the expression of *Prdm16* gene, both in vitro and in vivo.

### 3.3. Prdm16 Regulates the Expression of Ucp1 Gene in Brown Adipocytes

**Figure 3:**
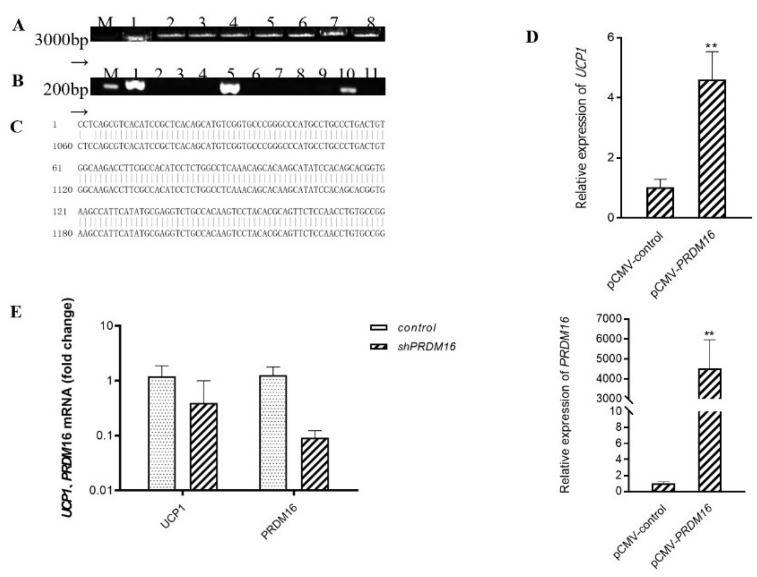
Overexpression of *Prdm16* upregulates *Ucp1* gene expression in brown adipose cells (**A**) Clone *Prdm16* electrophoretic map, M: Kang Wei 200B PLA DD; Lane 1–8: *Prdm16* amplification product. (**B**) PCR electrophoresis diagram of pCMV-Prdm16 bacterial solution; M: Kang Wei 200B PLA DD; Lane 1–11: PCR products from the picked bacterial solutions of 11 single colonies. (**C**) Comparative diagram of pCMV-Prdm16 sequencing results. (**D**) Effect of overexpression of *Prdm16* on *Ucp1* gene expression C3H10T12 cells. (**E**) Effects of shPRDM16 on Ucp1 gene expression during C_3_H_10_T_1/2_ cell differentiation. Compared with control, ** means *p* < 0.01.

We hypothesized accordingly that C3G may first upregulate the expression of gene *Prdm16* and then activate Ucp1. To explore and examine the effect of transcription factor *Prdm16* on *Ucp1* expression, we constructed a *p*CMV-Prdm16 vector to transfect C_3_H_10_T_1/2_ cells and further ascertained the level of *Ucp1* mRNA via quantitative real-time PCR after the completion of adipocyte differentiation (Figure 3A–C). As expected, *Prdm16* did regulate *Ucp1* expression at the level of transcription. In the treatment group in which *Prdm16* was overexpressed, the expression of *Ucp1* increased over four times more than that in the control group, with a significant up-regulation effect (Figure 3D) (*p* < 0.01). Further, to validate the loss-of-function studies, we constructed shPRDM16 to transfect C_3_H_10_T_1/2_ cells and further determine *Ucp1* mRNA levels by quantitative real-time polymerase chain reaction after adipocyte differentiation. As shown in Figure 3E, shPRDM16 reduced Ucp1 expression at the transcriptional level by more than 0.5 times that of the control group. Taken together, these results indicate that C3G did, at least partly, activate *Ucp1* by regulating the expression of the transcription factor *Prdm16*.

### 3.4. Prdm16 Directly Binds and Activates Ucp1 Promoter

**Figure 4:**
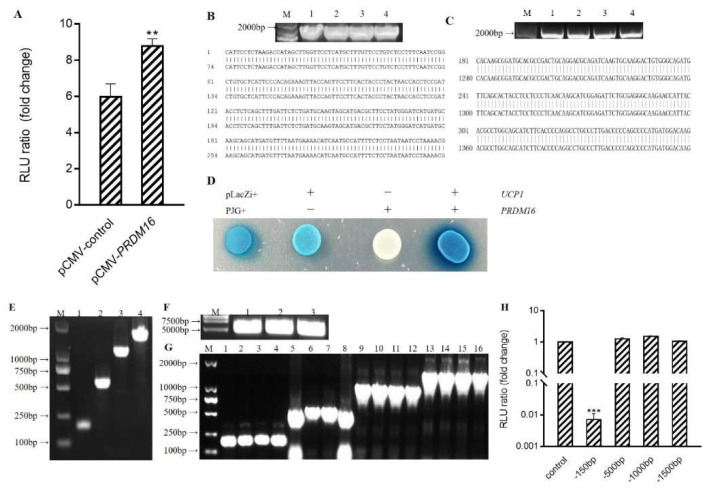
Prdm16 capable of activating *Ucp1* promoter (**A**) Effect of Prdm16 on *Ucp1* promoter (double luciferase report gene method) (**B**) PCR electrophoresis diagram and sequencing comparison diagram of pLacZi-Ucp1 bacterial solution. M: Kang Wei 200B PLA DD;Lane 1–4: PCR products from the picked four single-colony bacterial solutions. (**C**) PCR electrophoresis diagram and sequencing comparison diagram of pJG-Prdm16 bacterial solution. M: Kang Wei 200B PLA DD; Lane 1–4: PCR products from the picked four single-colony bacterial solutions. (**D**) Effect of Prdm16 on *Ucp1* promoter (yeast one-hybrid assay) (**E**) Clone electrophoretic images of Ucp1 promoter with different lengths. (**F**) PGL3-Basic enzyme digestion electrophoresis diagram (**G**) PCR electrophoresis map of bacterial solution after ligation of *Ucp1* promoter with different lengths to pGL3-Basic. (**H**) Effects of Prdm16 on promoters of different fragments *Ucp1*. Compared with control, ** means *p* < 0.01; *** means *p* < 0.001.

As a transcription factor, Prdm16 often regulates gene expression by binding to the promoter region of the target gene. To determine the mechanism by which Prdm16 regulates *Ucp1*, we performed a double luciferase reporter experiment to quantitate promoter activation. Compared to the empty vector control, co-transfection of *p*CMV-Prdm16 with the −2.0 kb *Ucp1* promoter–luciferase resulted in an obvious activation of the *Ucp1* promoter (Figure 4A) (*p* < 0.01).

This interaction of *Prdm16* with the *Ucp1* promoter was subsequently reverified. A *p*LacZi-Ucp1 reporter vector and *p*JG-Prdm16 fusion expression vector were constructed and transfected into yeast for blue-streak screening (Figure 4B–D). Similar to the positive control group of Plazzi+ and PJG+, treatment groups transfected with *p*LacZi-*Ucp1* and *p*JG-Prdm16 were blue and dark in color. The results verified by the single yeast hybrid assay showed that *Prdm16* could directly bind to the *Ucp1* promoter.

To start defining how Prdm16 activates the *Ucp1* promoter, 5′deletions of the *Ucp1* promoters driving a luciferase reporter were generated and co-transfected along with *p*CMV-Prdm16 into 293 cells (Figure 4E–G). All *Ucp1* promoter constructs deleted down to −500 bp, thus showing remarkable activation upon *Prdm16* co-transfection. However, *Ucp1* promoter activation by Prdm16 was lost when the promoter was deleted to −150 bp, indicating that Prdm16 functions through the sequence from −500 to −150 bp (Figure 4H).

### 3.5. PPARα Binds to Ucp1 Promoter to Activate Transcription

**Figure 5:**
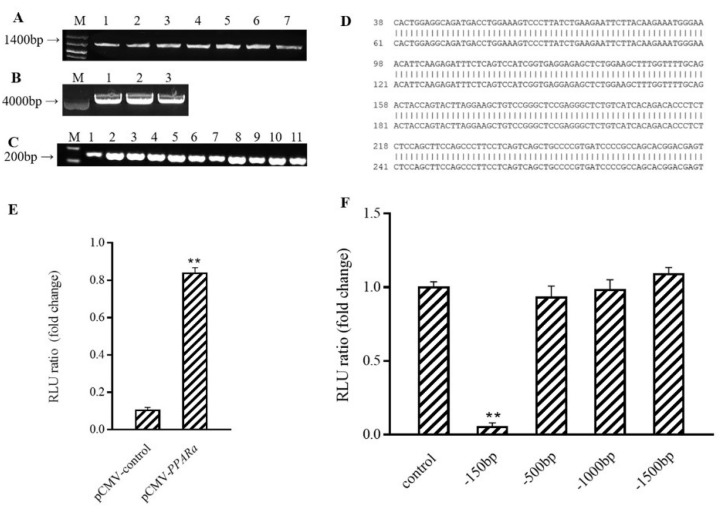
PPARα binds to *Ucp1* promoter to activate transcription. (**A**) Cloned PPARα electrophoresis M: Kang Wei 200B PLA DD; Lane 1–7: PPARα amplification product. (**B**) Electrophoresis map of pCMV-N-Flag enzyme digestion product, M: Kang Wei 200B PLA DD; Lane 1–3: pCMV-N-Flag double enzyme digestion product. (**C**) PCR electrophoresis diagram of pCMV-PPARα bacterial solution;M: Kang Wei 200B PLA DD; Lane 1–11: PCR products from picked 11 single-colony bacterial solutions (**D**) Comparison of pCMV-PPARα sequencing results. (**E**) Effect of PPARα on the intact *Ucp1* promoter (−2000 bp). (**F**) Effect of PPARα on promoter of *Ucp1* with different fragments. ** mesns *p* < 0.01.

The above experiments showed that Prdm16, regulated by C3G, plays a novel role in upregulating the expression of *Ucp1*; however, it has been previously reported that the expression of *Ucp1* is regulated by many factors. We found that *PPARα,* a gene associated with fatty acid oxidation, could also activate *Ucp1* promotion in the same way as Prdm16 (Figure 5A–E) (*p* < 0.01). In addition, the results of the luciferase activity assay showed that the activity of the −150 bp *Ucp1* promoter was significantly reduced (Figure 5F) (*p* < 0.01). Analysis, combined with the results of this assay, showed that the region where the *Ucp1* promoter binds to *PPARα* is between −500 and −150 bp upstream of its initiation site.

### 3.6. Conformational Investigation and Interaction Study of PPARα–C3G

**Figure 6:**
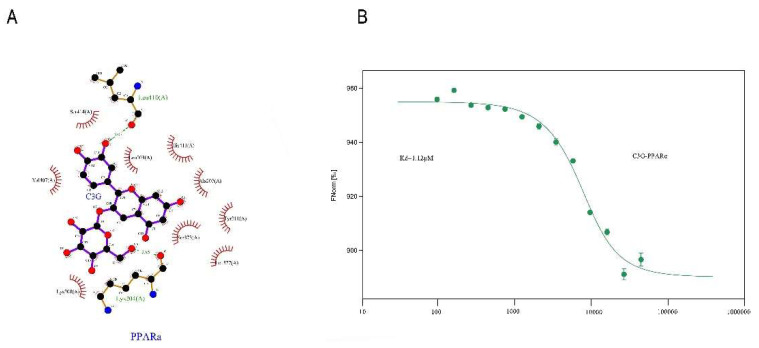
Conformational investigation and interaction study of PPARα–C3G (**A**).The molecular visualization system predicted the binding conformation of pparα to C3G. (**B**) MST technique to quantitate the affinity of pparα for C3G molecular interactions. The final C3G was added at varying concentrations (ranging from 5 μM to 23,000 μM).

Molecular modeling of the PyMOL program was used to improve understanding of the PPARα–C3G interaction. The optimal energy sequencing results for PPARα and C3G binding modes are shown in Figure 6A. As the thermophoresis law of molecules in solution is closely related to their molecular size, charge, and hydration layer properties, the change in any property will cause a change in its movement law, MST technology has extremely high sensitivity and wide application range. MST was utilized to further prove the interaction results by fluorescence spectroscopy. As shown in Figure 6B, the fitting curve of PPARα and C3G binding was well-shaped, with the Kd value of 1.12 μm. The Kdre value indicated the degree of dissociation of the complex in the equilibrium state. The smaller Kd value indicated that the degree of dissociation was lower, and the affinity between PPARα–C3G was stronger.

## 4. Discussion

This study proved, for the first time, that C3G does not upregulate the expression of Ucp1 by activating the *Ucp1* promoter. Another novel finding herein is that Prdm16 can bind to the region −500 to −150 bp upstream of the transcription start site of *Ucp1* gene, thereby activating the promoter activity. It was also proposed for the first time that C3G participates in the synthesis and thermogenesis of brown adipocyte Ucp1 by regulating *Prdm16*.

Thermogenesis is one of the most important functions of BAT, a tissue that has recently been shown to play a significant role in energy and glucose metabolism in adults, making it an interesting potential target for strategies aimed at augmenting the energy metabolic rate [23]. In short, increasing BAT activity may be a highly effective therapeutic approach to combating obesity and its related metabolic diseases.

Uncoupling proteins (UCPs) are a class of heat-producing proteins distributed on the inner membrane of mitochondria. Their activity is closely related to heat loss, regulated by purine nucleosides (ADP, ATP), guanosine (GDP, GTP), and free fatty acids (FFA), and they, therefore, play an important role in mammalian heat production and energy metabolism regulation [24]. There are five known main types of UCPs, namely Ucp1, UCP2, UCP3, UCP4, and UCP5. Ucp1 activity exists in mammalian BAT, while UCP2 is widely distributed in whole body tissues, such as white adipose tissue (WAT), heart, liver, kidney, spleen, thyroid, and gastrointestinal tract [25]. UCP3 is mainly distributed in skeletal muscle and UCP4 is mainly distributed in brain [26,27], while UCP5 is mainly found in brain and neural tissues [28]. Ucp1 is the only expression of uncoupling protein found in BAT.

Brown fat cells are characterized by densely packed mitochondria that contain Ucp1 in their inner mitochondrial membrane [29]. BAT thermogenesis is dependent on Ucp1 as it is a proton transporter that allows protons to leak across the mitochondrial inner membrane, thereby dissipating the electrochemical gradient normally used for ATP synthesis [29]. The regulation of levels of the *Ucp1* gene, thus, plays a decisive role in the control of Ucp1 content and is at the center of the physiological regulation of BAT heat production [30]. Revealing the involved signal pathway and transcription regulation of Ucp1 in BAT’s energy metabolic process not only helps us to better understand the important role of Ucp1 in BAT energy metabolism control but also provides a theoretical foundation for obesity treatment based on BAT.

Our previous research showed that dietary supplementation with the functional ingredients of natural plants exhibited a positive effect on BAT functionality in mice. Such plant-based ingredients include mulberry polyphenol extract, such as C3G and rutin [4,18,31]. Moreover, some phenolic acids, such as vanillic acid and chlorogenic acid, which are components of mulberry fruit polyphones, also induce the thermogenesis of BAT and could, therefore, be applied to the prevention of dietary-induced obesity and insulin resistance [31,32]. We previously reported that mulberry and mulberry wine extract (ME and MWE) can not only improve the expression of BAT specific gene *Ucp1*, but also improve the mitochondrial copy number [33]. As the most abundant anthocyanin in ME and MWE, C3G has also been proven to increase the quantity of mitochondria in subsequent experiments [17]. In order to further explore the heat production mechanism of BAT promoted by C3G, we studied the mitochondrial biogenesis and function. The results show that C3G significantly upregulated the expression of mitochondrial synthesis-related genes *Tfam, NRF1* and *NRF2*, in both the BAT of both the db/db and mice with DIO, and increased the number of mitochondria and the expression levels of mitochondrial oxidative phosphorylation-related proteins ATP5A, UQCR2, and NDUFB8 [4,18].

This strongly suggested the promotion of BAT heat production by C3G, thus warranting further exploration of the mechanism by which C3G regulates the action of *Ucp1* and its related heat production gene. In this study, we first verified the promotional effect of C3G on *Ucp1* gene expression through animal models. Thereafter, we found that C3G can upregulate the BAT specific gene *Ucp1* expression during brown adipocyte differentiation, thereby effectively increasing the mitochondrial respiration uncoupling of brown adipocytes, which is consistent with the previously reported conclusion [34]. However, our study found that there is no concentration dependence, and also that the activation of *Ucp1* by C3G is not achieved by activating the promoter, thus suggesting the existence of other regulatory factors in the process.

Numerous studies have shown that the activity of Ucp1 is inhibited by nucleotides and activated by non-lipidated fatty acids [33,35]. Norepinephrine (NE) released by sympathetic nerves or hypothermia are the main physiological signals that activate Ucp1 synthesis in BAT cells [36]. Furthermore, the synthesis of Ucp1 is strongly regulated by the transcription level; cAMP is the main activator of *Ucp1* gene transcription [37,38,39,40]; and β3-AR-specific agonists L-755507, CGP12177, CL316243, and BRL35135 can significantly increase the levels of *Ucp1* mRNA in mammalian BAT cells [38,39,40]. In addition, several transcription factors and coregulators found in both WAT and BAT, as well as in other tissues, including PPARs, PGC1α, and ATF2, have been implicated in the transcriptional activation of *Ucp1* [12,41]. Our data show that C3G can activate *Ucp1* by upregulating the expression of the transcription factor *Prdm16*. Since Prdm16 is related to heat production [42,43], it is even clearer that C3G does indeed play a role in weight loss by activating heat production.

Prdm16 can form transcription complexes with other transcription factors, such as C/EBPβ, PGC1α, PPARα, and PPARγ, to regulate the expression of thermogenic genes [14,44]. Furthermore, it was found in our previous research that C3G can upregulate PPARα [4]. Consequently, on the basis of the above discussion, it would be reasonable to believe that PPARα is also involved in *Ucp1* transcriptional regulation. Through double luciferase experiments, we ascertained that PPARα can indeed activate the transcriptional expression of *Ucp1* in a similar manner to that of Prdm16. Unlike Prdm16, however, PPARα is a fatty acid oxidation-related gene that is also expressed in WAT, which suggests that the transcriptional activation of C3G to *Ucp1* is not limited to BAT. C3G is expected to achieve weight loss and lipid reduction by increasing WAT heat production, which would be consistent with previous conclusions [18].

On the one hand, we proved that C3G could enhance the thermogenesis of BAT. On the other hand, anthocyanins were reported to have anti-cancer and anti-inflammatory effects [45], which were closely related to their antioxidant activities. Therefore, the role of antioxidant activity of C3G in thermogenesis could be further explored. However, there are a variety of metabolites of C3G, and 41 metabolites have been identified. Among them, protocatechuic acid and vanilloid are metabolites that exert the main physiological functions of C3G in the body [46]. For example, PCA and PGA, the degradation products of C3G, inhibit the proliferation of cancer cells, which is closely related to their antioxidant activity [47]. The effect of C3G on the thermogenesis of brown fat suggests that its metabolites may have the same effect or even more obvious effects. Therefore, the above C3G metabolites can be further studied in future studies to further clarify the effective activators that activate *Ucp1*.

## 5. Conclusions

Taken together, the findings of our study indicate that C3G can upregulate the expression level of Prdm16, and then activate the transcriptional expression of *Ucp1* by directly binding to the −500 to −150 bp region of the *Ucp1* promoter.

In future research, it would be valuable to determine whether Prdm16 interacts with PPARα or other activating factors to activate *Ucp1* and to determine the molecular mechanism by which C3G regulates Prdm16 expression. The results of this study provide an effective approach for the development of novel methods to improve energy metabolism with the activation of Ucp1 as the target, and also help to establish an innovative theoretical basis for the in-depth utilization and development of C3G-rich fruit and vegetable resources.

## Figures and Tables

**Table 1:** Primer sequences used for qRT-PCR.

Gene	Primer Sequence (5′→3′)
*Ucp1*	F:GGCAAAAACAGAAGGATTGC R:TAAGCCGGCTGAGATCTTGT
*Prdm16*	F:GAAGTCACAGGAGGACACGGR: CTCGCTCCTCAACACACCTC

**Table 2:** The cleavage sites and vector plasmids of the different gene fragments.

Vector	Gene	Primer Sequence (5′→3′)
Double luciferase reporter gene vector of *Ucp1* promoter	*Ucp1-KPN I-F*	GGGGTACCcattcctctaagaccatagctt
	*Ucp1-Mlu* *Ⅰ* *-R*	CGACGCGTacttctgcgccctgacct
Eukaryotic vector for overexpression of *Prdm16*	*Prdm16-EcoR* *I-F*	tccaagcttctgcaggaattcA TGCGA TCCAAGGCGAGG
	*Prdm16-Xba* *I-R*	accgggcccactagttctagaTCA TTGCA TA TGCCTCCGGG
Double luciferase reporter gene vector of *Ucp1* promoter with different fragment size	*Ucp1(-150bp)-Kpn I-F*	atttctctatcgataggtaccGAGTGACGCGCGGCTGGG
	*Ucp1(-150bp)-Mlu I-R*	cgagcccgggctagcacgcgtCTGCGCCCTGACCTGGGA
	*Ucp1(-500bp)-Kpn I-F*	atttctctatcgataggtaccTCCAGTCACCCAAA TCTGAAGG
	*Ucp1(-500bp)-Mlu I-R*	cgagcccgggctagcacgcgtCTGCGCCCTGACCTGGGA
	*Ucp1(-1000bp)-Kpn I-F*	atttctctatcgataggtaccAGCAGAACCTGGCCAACCA
	*Ucp1(-1000bp)-Mlu I-R*	cgagcccgggctagcacgcgtCTGCGCCCTGACCTGGGA
	*Ucp1(-1500bp)-Kpn I-F*	atttctctatcgataggtaccTTATTATACTGTTGTTGCTGCTGCT
	*Ucp1(-1500bp)-Mlu I-R*	cgagcccgggctagcacgcgtCTGCGCCCTGACCTGGGA
pLacZi-Ucp1	*Ucp1-EcoR I-F*	ctttgatattggatcgaattcCATTCCTCTAAGACCATAGCTTGGT
	*Ucp1-Xho I-R*	atacagagcacatgcctcgagACTTCTGCGCCCTGACCTG
	*Prdm16-EcoR I-F*	gattatgcctctcccgaattcTACGCTAGGTTCCGCTCCC
	*Prdm16-Xho I-R*	agaagtccaaagcttctcgagTAGTAACGTA TACGGAGGCCCA T
PJG-Prdm16	*Prdm16-EcoR I-F*	gattatgcctctcccgaattcTACGCTAGGTTCCGCTCCC
	*Prdm16-Xho I-R*	agaagtccaaagcttctcgagTAGTAACGTA TACGGAGGCCCA T

## Data Availability

The data presented in this study are available in this manuscript.
